# Vimentin Phosphorylation Is Required for Normal Cell Division of Immature Astrocytes

**DOI:** 10.3390/cells8091016

**Published:** 2019-09-01

**Authors:** Yolanda de Pablo, Pavel Marasek, Andrea Pozo-Rodrigálvarez, Ulrika Wilhelmsson, Masaki Inagaki, Marcela Pekna, Milos Pekny

**Affiliations:** 1 Laboratory of Astrocyte Biology and CNS Regeneration, Center for Brain Repair, Department of Clinical Neuroscience, Institute of Neuroscience and Physiology, Sahlgrenska Academy at the University of Gothenburg, 40530 Gothenburg, Sweden; 2 Laboratory of Regenerative Neuroimmunology, Center for Brain Repair, Department of Clinical Neuroscience, Institute of Neuroscience and Physiology, Sahlgrenska Academy at the University of Gothenburg, 40530 Gothenburg, Sweden; 3 Department of Physiology, Mie University Graduate School of Medicine, Mie 5148507, Japan; 4 Florey Institute of Neuroscience and Mental Health, Parkville, Victoria 3052, Australia; 5 University of Newcastle, New South Wales 2308, Australia

**Keywords:** intermediate filaments, nanofilaments, vimentin, vimentin accumulations, GFAP, astrocytes, immature astrocytes, mitochondria

## Abstract

Vimentin (VIM) is an intermediate filament (nanofilament) protein expressed in multiple cell types, including astrocytes. Mice with *VIM* mutations of serine sites phosphorylated during mitosis (*VIM^SA/SA^*) show cytokinetic failure in fibroblasts and lens epithelial cells, chromosomal instability, facilitated cell senescence, and increased neuronal differentiation of neural progenitor cells. Here we report that in vitro immature *VIM^SA/SA^* astrocytes exhibit cytokinetic failure and contain vimentin accumulations that co-localize with mitochondria. This phenotype is transient and disappears with *VIM^SA/SA^* astrocyte maturation and expression of glial fibrillary acidic protein (GFAP); it is also alleviated by the inhibition of cell proliferation. To test the hypothesis that GFAP compensates for the effect of *VIM^SA/SA^* in astrocytes, we crossed the *VIM^SA/SA^* and *GFAP^−/−^* mice. Surprisingly, the fraction of *VIM^SA/SA^* immature astrocytes with abundant vimentin accumulations was reduced when on *GFAP^−/−^* background. This indicates that the disappearance of vimentin accumulations and cytokinetic failure in mature astrocyte cultures are independent of GFAP expression. Both *VIM^SA/SA^* and *VIM^SA/SA^GFAP^−/−^* astrocytes showed normal mitochondrial membrane potential and vulnerability to H_2_O_2_, oxygen/glucose deprivation, and chemical ischemia. Thus, mutation of mitotic phosphorylation sites in vimentin triggers formation of vimentin accumulations and cytokinetic failure in immature astrocytes without altering their vulnerability to oxidative stress.

## 1. Introduction

Cytoplasmic intermediate filaments (also known as nanofilaments) of astrocytes are highly dynamic structures involved in cell signaling and cell migration and act as a signaling platform which controls cell responses in health, disease, and during stress and regeneration responses [1,2,3,4]. Astrocyte intermediate filaments play a key role in astrocyte activation [4,5,6,7] and astrocyte response to central nervous system injury called reactive gliosis; upregulation of intermediate filament proteins such as glial fibrillary acidic protein (GFAP), vimentin, and nestin is an important cellular hallmark of reactive gliosis. Data from mice lacking GFAP and vimentin (*GFAP^−/−^Vim^−/−^*) and other experimental modulations of reactive gliosis point to reactive gliosis as protective in ischemic stroke [4,7,8,9], neurotrauma [5,10,11,12], and neurodegenerative diseases [13,14,15]. However, in some disease situations, reactive gliosis inhibits later regenerative responses [6,16,17]. *GFAP^−/−^Vim^−/−^* mice, which have no cytoplasmic astrocyte intermediate filaments and show attenuated reactive gliosis [18,19], exhibit better regeneration of synapses and axons after trauma [5,20], improved recovery after spinal cord trauma [21], reduced retinal degeneration [22], and better integration of retinal grafts [16] and transplanted neural stem cells [17]. These results show that, in some pathological conditions, the benefits of reactive gliosis that are manifest acutely after injury correlate inversely with regenerative potential and recovery at later stages and point to astrocyte intermediate filaments as a potential target for therapies of neurological diseases.

Phosphorylation of serine/threonine residues in the head domain of intermediate filament proteins regulates the disassembly of intermediate filaments [23,24,25,26,27,28] and is essential for cell division [29,30,31,32,33]. The key vimentin phosphorylation sites and the protein kinases involved are known [29,30,31,34,35,36,37,38,39,40,41,42,43,44], and the mice with all eleven vimentin serines that are phosphorylated during mitosis substituted by alanine (*VIM^SA/SA^* mice) age prematurely, develop cataract, and show progressive loss of fat and impaired healing of skin wounds [45,46]. *VIM^SA/SA^* fibroblasts and lens epithelial cells exhibit cytokinetic failure, aneuploidy, chromosomal instability, and increased expression of markers of cell senescence [45]. *VIM^SA/SA^* mice show an increase in the fraction of newly born and surviving neurons in the dentate gyrus of the hippocampus, one of the two adult neurogenic zones. *VIM^SA/SA^* neurosphere cells exhibit several-fold increased neuronal differentiation; this effect of *VIM^SA/SA^* mutation is neurosphere cell-autonomous, and not caused by co-cultured astrocytes [47]. Mature *VIM^SA/SA^* astrocytes in culture show normal cell morphology and proliferation with a normal rate of cytokinetic failure, well-developed network of intermediate filaments despite downregulation of vimentin and upregulation of GFAP, and they are as capable as wild-type mature astrocytes to close in vitro wounds [47].

In the current study, we investigated the effects of *VIM^SA/SA^* in immature astrocytes that express lower levels of GFAP. In addition, we addressed potential compensatory effects of GFAP in *VIM^SA/SA^* astrocytes by generating the *VIM^SA/SA^GFAP^−/−^* mice.

## 2. Materials and Methods

### 2.1. Animals

In *VIM^SA/SA^* mice, the 11 serines phosphorylated during mitosis were replaced by alanine [45]. *VIM^SA/SA^* mice were on C57Bl/6 genetic background. *GFAP^−/−^* mice were generated as described before [48]. Mice carrying both the *VIM^SA/SA^* and the *GFAP^−/−^* mutations were on a mixed C57Bl6/129Sv/129Ola genetic background. C57Bl/6 or mixed genetic background wild-type mice were used as control groups as appropriate. Mice were kept in standard cages in a barrier animal facility with free access to food and water. All experiments were approved by the Ethics Committee of the University of Gothenburg (2018-05-16; diary number 1551/2018).

### 2.2. Antibodies

**Figure 1:**
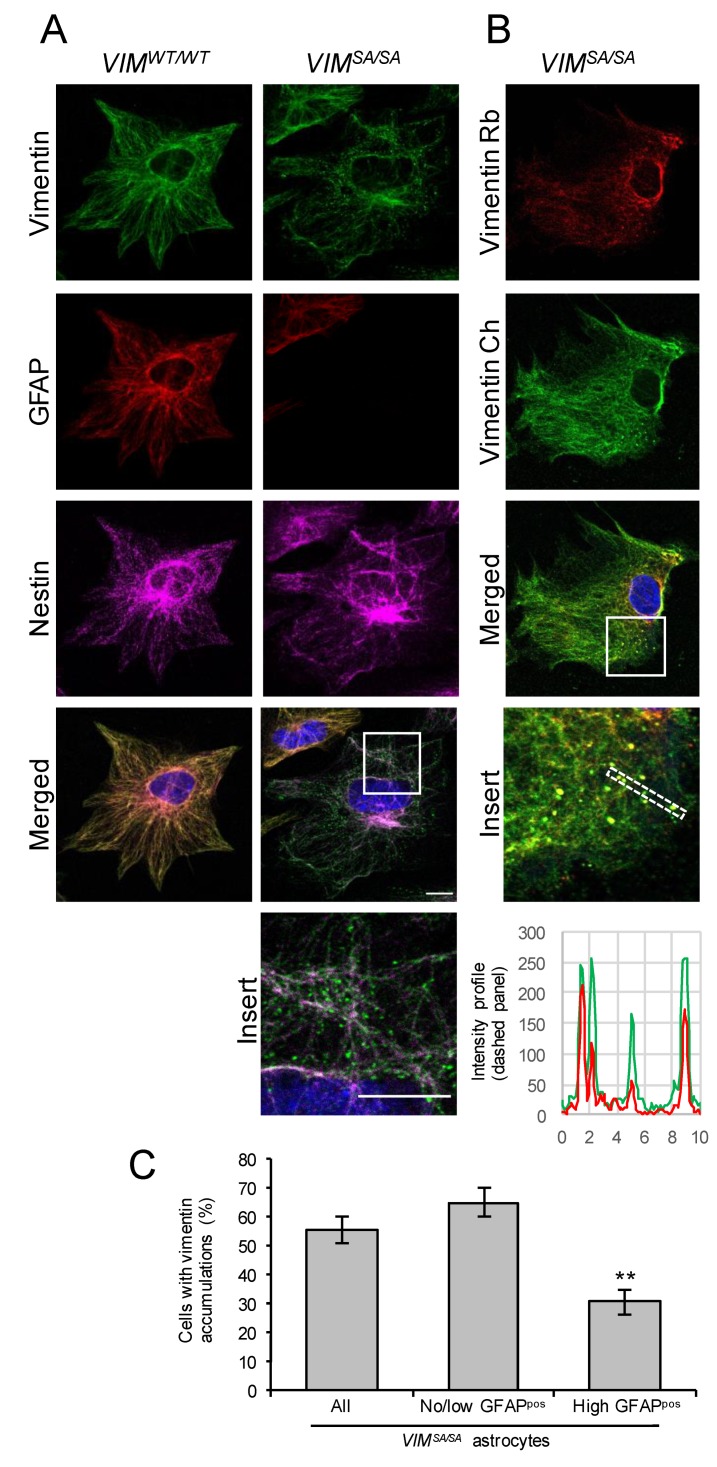
Immature *VIM^SA/SA^* astrocytes contain vimentin accumulations. (**A**) Immature *VIM^WT/WT^* and *VIM^SA/SA^* astrocytes were labeled with antibodies against vimentin (green), glial fibrillary acidic protein (GFAP) (red), and nestin (purple). Nuclei were visualized with DAPI (blue). Vimentin accumulations were absent in *VIM^WT/WT^*, but clearly visible in immature *VIM^SA/SA^* astrocytes, in particular in cells with low or no GFAP expression. (**B**) Immature *VIM^SA/SA^* astrocytes were labeled with rabbit (Rb) or chicken (Ch) polyclonal antibodies against vimentin. Vimentin accumulations were detected by both antibodies (see also the intensity profiles). (**C**) The fraction of vimentin accumulations containing astrocytes among all *VIM^SA/SA^* astrocytes (left bar) and among astrocytes with high expression of GFAP (right bar). N = 6 mice; for each mouse, on average 560 astrocytes in total and 156 GFAP highly positive astrocytes, respectively, were evaluated. Scale bar 10 µm, ** *p* < 0.01.

Rabbit anti-nestin (for immunofluorescence 1:2500, for western blot 1:2000; BioLegend (San Diego, CA, USA, 839801), mouse anti-GFAP (for immunofluorescence 1:100; Merck (Darmstadt, Germany), MAB360; for western blot 1:250; Dako (Glostrup, Denmark), M0761), chicken anti-vimentin (for immunofluorescence 1:1000; used throughout the study; for western blot 1:2000; BioLegend, 919101), rabbit anti-vimentin (1:200; Abcam (Cambridge, UK), ab45939; used for the comparison in Figure 1), rabbit anti-TOMM20 (1:200; Abcam, ab186734), mouse anti-Ki67 (1:50, BD Biosciences (Franklin Lakes, NJ, USA, 550609), goat anti-chicken Alexa Fluor 488 (1:1000; Thermo Fisher Scientific, (Waltham, MA, USA, A11039), donkey anti-mouse Alexa Fluor 555 (1:1000; Thermo Fisher Scientific, A31570), donkey anti-rabbit Alexa Fluor 647 (1:1000; Thermo Fisher Scientific, A31573), donkey anti-rabbit Alexa Fluor 555 (1:1000; Thermo Fisher Scientific, A31572), rabbit anti-GAPDH–HRP conjugate (1:500; Cell Signaling Technology, (Beverly, MA, USA, 3683), goat anti-rabbit-HRP conjugate (1:1000; Cell Signaling Technology, 7074), and horse anti-mouse-HRP conjugate (1:1000; Cell Signaling, 7076) were used. The specificity of the GFAP, vimentin, and nestin antibodies was previously validated, on tissues/cell cultures from mice carrying null mutations in the respective genes serving as negative controls.

### 2.3. Astrocyte Cultures

Astrocyte-enriched cultures were prepared from the brain cortex of postnatal day 2 mice as previously described [7]. The cell suspension was seeded on a poly-d-lysine-coated flask. Cells were grown in DMEM supplemented with 10% FBS, 2 mM l-glutamine, 1% penicillin/streptomycin and kept at 37 °C in a humidified atmosphere with 5% CO_2_. For preparation of immature astrocytes, cells were seeded on a 25 cm^2^ flask (one mouse/flask) and passaged after five days.

### 2.4. Immunofluorescence

**Figure 2:**
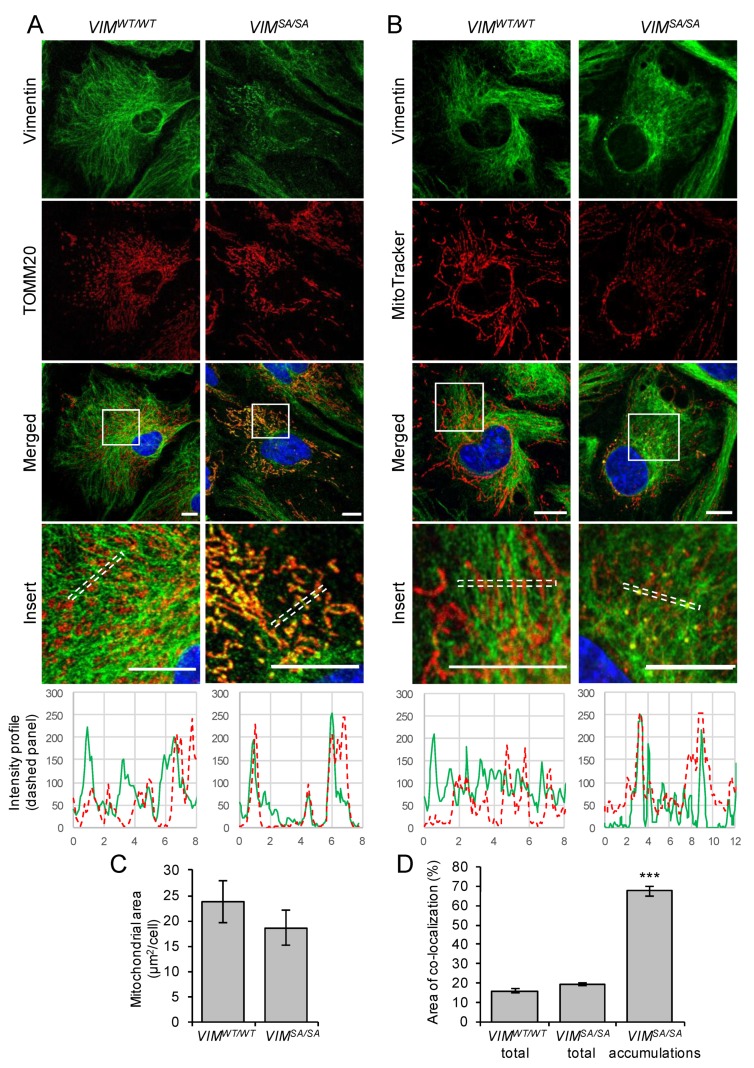
Vimentin accumulations co-localize with mitochondria. Immature *VIM^SA/SA^* astrocytes were labeled with polyclonal antibodies against vimentin (green) and the mitochondrial protein TOMM20 (red, **A**) or the fluorescent mitochondrial probe MitoTracker (red, **B**). Nuclei were visualized with DAPI (blue). (**C**). Mitochondrial area was measured from MitoTracker images. (**D**) The area of vimentin co-localization with MitoTracker was measured for total vimentin immunofluorescence and vimentin accumulations. N = 11 and 12 per genotype. Scale bar 10 µm. *** *p* < 0.001

**Figure 3:**
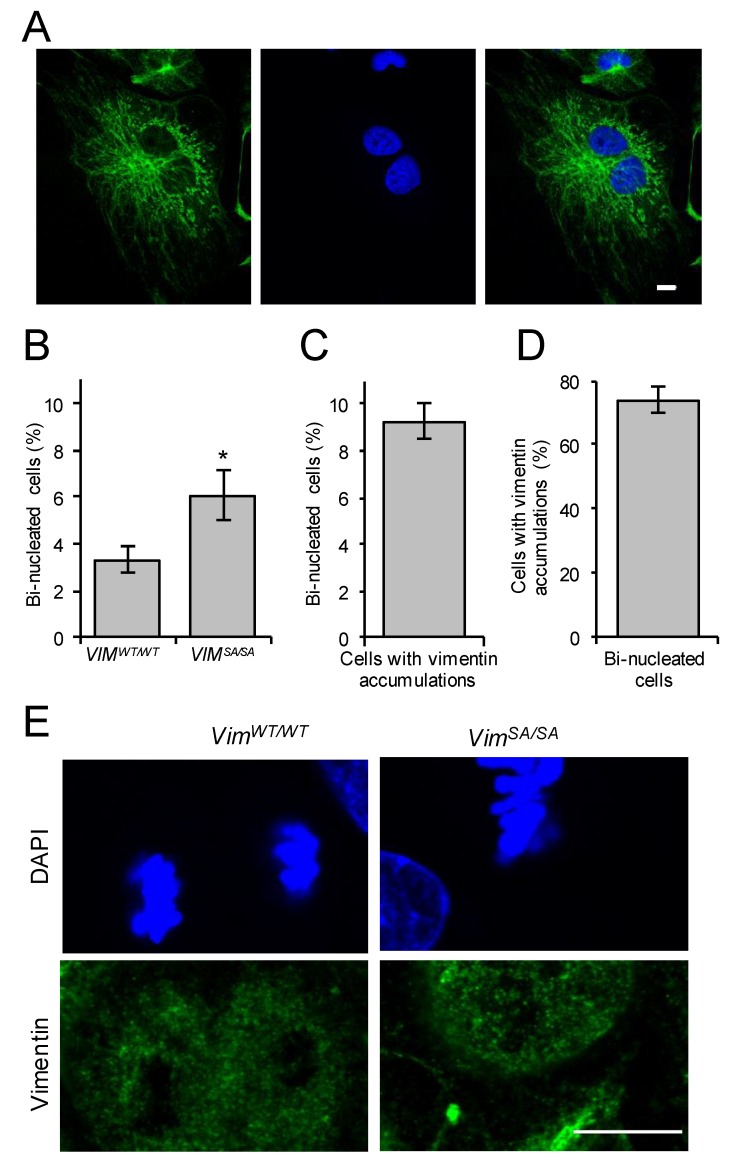
Increased fraction of bi-nucleated cells among immature *VIM^SA/SA^* astrocytes. Immature *VIM^WT/WT^* and *VIM^SA/SA^* astrocytes were labeled with polyclonal antibodies against vimentin (green) and nuclei were visualized with DAPI (blue). Images were taken and bi-nucleated cells were counted. (**A**) Representative example of a bi-nucleated cell. (**B**) Fraction of bi-nucleated cells among immature *VIM^WT/WT^* and *VIM^SA/SA^* astrocytes, on average 100 cells were assessed for each mouse, N = 11 and 12, respectively. * *p* < 0.05. (**C**) Fraction of bi-nucleated cells among immature *VIM^SA/SA^* astrocytes with vimentin accumulations. (**D**) Fraction of cells with vimentin accumulations among bi-nucleated cells. In **C** and **D**, 225 cells were assessed on average, *n* = 6. (**E**) Examples of mitotic cells with disassembled vimentin intermediate filaments. Scale bars, 10 µm.

**Figure 4:**
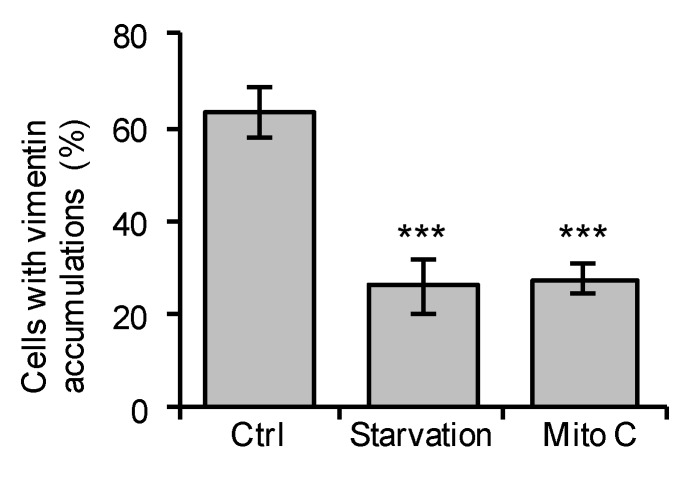
Serum starvation or mitomycin C treatment reduces the fraction of immature *VIM^SA/SA^* astrocytes containing vimentin accumulations. Immature astrocytes were cultured for three days in normal media (Ctrl), one day in normal media followed by two days in media without FBS (Starvation), or two days in normal media followed by 4 h exposure to mitomycin C (10 µg/m) then one day in normal media (Mito C). Cells were fixed and vimentin was visualized by immunofluorescence. On average, 460 cells were evaluated for each condition. N = 6 per group, *** *p* < 0.001.

**Figure 5:**
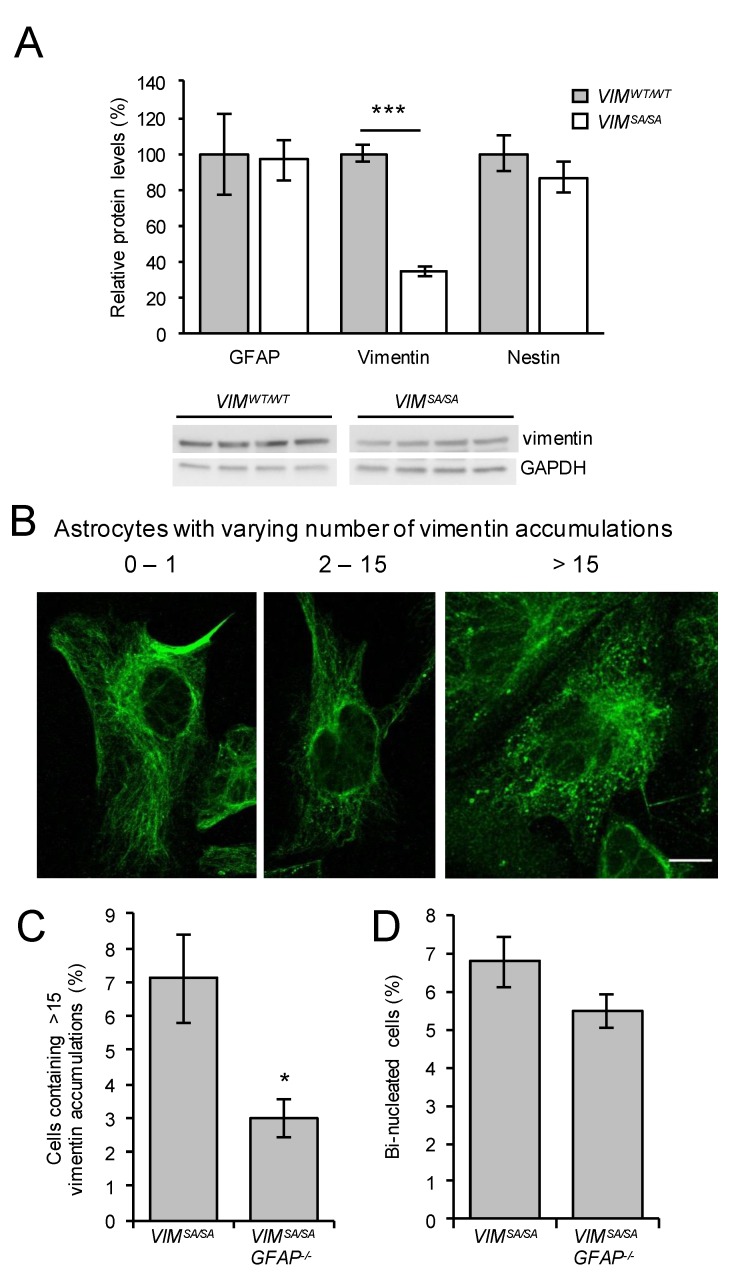
The fraction of immature *VIM^SA/SA^* astrocytes with abundant vimentin accumulations is reduced in the absence of GFAP. (**A**) Immature *VIM^WT/WT^* and *VIM^SA/SA^* astrocyte cultures were lysed and total protein levels of GFAP, vimentin, and nestin were compared by western blot analysis (normalized to GAPDH). Vimentin levels were lower in immature *VIM^SA/SA^* astrocytes. N = 4 per genotype; *** *p* < 0.001. (**B**–**D**) Immature *VIM^SA/SA^* and *VIM^SA/SA^GFAP^−/−^* astrocyte cultures were fixed, and vimentin was detected by immunofluorescence. (**B**) Examples of astrocytes with varying number of vimentin accumulations. (**C**) Astrocytes containing >15 vimentin accumulations were less abundant in *VIM^SA/SA^GFAP^−/−^* compared to *VIM^SA/SA^* cultures. (**D**) The fraction of bi-nucleated cells was comparable between *VIM^SA/SA^* and *VIM^SA/SA^GFAP^−/−^* astrocytes. N = 6 per genotype; * *p* < 0.05. Scale bar 10 µm.

Astrocytes were passaged onto laminin-coated glass coverslips (25,000–40,000 cells/cm^2^) and further incubated for two days. Cells were washed with PBS, fixed in 4% paraformaldehyde in PBS, and subsequently permeabilized with 0.1% Triton X-100/PBS (Sigma-Aldrich). Cells were then incubated with primary antibodies at room temperature for 1 h, followed by incubation with fluorophore-conjugated secondary antibodies for 1 h. Cells were washed three times for 5 min with PBS containing 0.05% Tween in between incubations. Cell nuclei were counterstained with DAPI (Sigma-Aldrich, St. Louis, MO, USA). For labeling of mitochondria with MitoTracker (Thermo Fisher Scientific, M7512), astrocytes were incubated for 15 min under standard culture conditions with 8 ng/mL MitoTracker followed by two washes in PBS and immediately fixed with ice cold methanol for 5 min at −20 °C and processed for immunofluorescence as described. Labeled cells were imaged using an LSM 700 (Figures 1, 2A,B, 4, and 5) and LSM 780 (Figures 2C,D and 3) confocal microscopes (Zeiss, Oberkochen Tyskland) with sequential scanning for the four channels. Intensity profiles were obtained using ImageJ software [49,50] on RGB images (a line selection at the center of the dashed panel as shown in Figure 2). All images within a given experiment were acquired with the same settings for each fluorophore. Scale bars, brightness/contrast adjustments, and image type transformation were performed using ImageJ. For statistical analysis of cells containing vimentin accumulations and bi-nucleated cells, the coverslips were scanned with scanR high-content microscope (Olympus, Shinjuku, Japan) and analyzed using scanR analysis software. The automatic analysis was used for counting the total cell number, and identifying GFAP strongly positive cells (threshold 600). The number of cells containing vimentin accumulations and bi-nucleated cells was determined manually. Co-localization between vimentin and mitochondria was quantified using ImageJ. In brief, mitochondrial and total vimentin areas were quantified using the algorithm auto threshold moments dark and the area occupied by vimentin accumulations was quantified using the algorithm auto threshold intermodes dark, size 0.01–10, minimum circularity 0.9.

### 2.5. Western Blot

Immature astrocytes were collected by trypsinization at seven days in culture, washed twice with DPBS and lysed in RIPA buffer (20 mM Tris-HCl, pH 7.5, 150 mM NaCl, 1 mM EDTA, 1 mM EGTA, 0.1% SDS, complete protease inhibitor cocktail (Roche, Basel, Switzerland)). Lysates were sonicated 3 × 10 s to shear DNA, adjusted to the same protein concentration (Bio-Rad DC protein assay, Bio-Rad, Hercules, CA, USA), mixed with SDS sample buffer and boiled for 5 min. Before loading on 4–20% gradient gels (Mini-PROTEAN TGX, Bio-Rad), lysates were spun down at 16,000 *g*, for 20 min to pellet any insoluble material. Separated proteins were transferred onto a PVDF membrane (0.45 µm; Millipore/Immobilon), which was subsequently blocked in 1% skimmed milk and incubated with appropriate primary and HRP-conjugated secondary antibodies. The signal was developed using ECL western blotting detection reagents (GE Healthcare, Chicago, IL, USA) and the membrane was scanned on a LAS-3000 image analyzer (Fujifilm, Minato, Japan).

### 2.6. Mitochondrial Membrane Potential Measurement

Immature astrocytes were seeded at a density of 70,000 cells/well on poly-D-lysine-coated 24-well plates. Cells were washed and incubated in PBS for 2 h followed by loading with TMRE (Sigma-Aldrich, 87917) 10 nM in HBSS (Hank’s Balanced Salt Solution) during 30 min. Cells were washed in HBSS and fluorescence was measured in a SpectraMax ID3 plate reader (Molecular devices, San Jose, CA, USA) at 530/580 nm. Cultures were fixed and stained with Coomassie brilliant blue; absorbance was measured for normalization to total protein.

### 2.7. Cell Death Measurement

Immature astrocytes were seeded at a density of 70,000 cells/well on poly-D-lysine-coated 24-well plates. Treatments were started at day 2 after passaging. Chemical ischemia was induced by NaN_3_ (2.5 mM) and 2-deoxy-glucose (2 mM) in PBS. For oxygen/glucose deprivation (OGD), astrocytes were incubated in OGD buffer (51 mM NaCl, 65 mM K-gluconate, 0.13 mM CaCl_2_, 1.5 mM MgCl_2_ and 10 mM HEPES pH 6.8, penicillin and streptomycin) as described previously [7,51]. Lactate dehydrogenase (LDH) was measured in the supernatant following the manufacturer’s instructions (Takara Bio, Shiga, Japan, MK401).

### 2.8. Data Analysis

Statistical analyses were performed using Excel (Microsoft, Redmond, WA, USA). Two-tailed *t* test was used for comparison between two groups assuming equal variance. Differences were considered significant at *p* < 0.05. All values were presented as mean ± SEM.

## 3. Results

### 3.1. Immature VIM^SA/SA^ Astrocytes Contain Vimentin Accumulations

*VIM^SA/SA^* astrocytes cultured for 14 days show normal vimentin intermediate filament network and no signs of mitotic failure [47]. In order to detect any possible alteration in intermediate filament network formation, we assessed the vimentin network morphology in immature *VIM^SA/SA^* astrocytes cultured for seven days (Figure 1). As in wild-type (*VIM^WT/WT^*) astrocytes, vimentin immunoreactivity in immature *VIM^SA/SA^* astrocytes was present in the form of well-developed intermediate filament bundles. However, in more than 50% of cells, we found vimentin immunoreactivity that exhibited a punctate pattern (that we henceforth call vimentin accumulations) and that was absent in *VIM^WT/WT^* astrocytes. The same pattern was observed by using two different antibodies against vimentin (Figure 1B). These vimentin accumulations appeared predominantly in immature *VIM^SA/SA^* astrocytes with few or no GFAP intermediate filament bundles (*p* = 0.0018, Figure 1C), rather than in immature *VIM^SA/SA^* astrocytes with abundant GFAP intermediate filament bundles (the latter constituted about 27% of all astrocytes).

### 3.2. Vimentin Accumulations in Immature VIM^SA/SA^ Astrocytes Co-Localize with Mitochondria

Given that intermediate filament proteins such as neurofilament proteins [52], vimentin [53,54], desmin [55], and keratins [56] were reported to affect mitochondrial intracellular localization and shape, we investigated the co-localization of vimentin and mitochondria in immature astrocytes. Mitochondria were visualized using antibodies against the outer mitochondrial membrane protein TOMM20 (Figure 2A) and by using the mitochondrial probe MitoTracker, which is dependent on mitochondrial membrane polarization (Figure 2B). The abundance of mitochondria was not changed in *VIM^SA/SA^* astrocytes (*p* = 0.351, Figure 2C). While the co-localization of vimentin intermediate filament bundles with mitochondria was low both in *VIM^WT/WT^* and *VIM^SA/SA^* astrocytes (<20%; *p* = 0.052), the co-localization of vimentin accumulations and mitochondria in *VIM^SA/SA^* compared to *VIM^WT/WT^* astrocytes was strikingly high (67.5%, *p* < 0.00000001, Figure 2D).

### 3.3. Increased Fraction of Bi-Nucleated Cells among Immature VIM^SA/SA^ Astrocytes

In lens epithelial cells, *VIM^SA/SA^* leads to chromosomal instability, including bi-nucleation and aneuploidy [45]. In mature astrocytes cultured for 14 days there was no difference in the numbers of bi-nucleated cells between *VIM^WT/WT^* and *VIM^SA/SA^* cultures [47]. However, the number of bi-nucleated cells (Figure 3A) in immature *VIM^SA/SA^* astrocyte cultures was higher than that in *VIM^WT/WT^* astrocytes (6.1 ± 1% and 3.3 ± 0.5%, respectively, *p* = 0.036; Figure 3B), and the fraction of bi-nucleated cells in the subpopulation of astrocytes containing vimentin accumulations was 9.2 ± 0.8% (Figure 3C). The observation of about 75% of bi-nucleated immature *VIM^SA/SA^* astrocytes (74.0 ± 3.8%) containing vimentin accumulations (Figure 3D) links *VIM^SA/SA^* to cytokinetic failure. Immature astrocytes undergoing mitosis with a disassembled intermediate filament network were detected in cultures of both *VIM^WT/WT^* and *VIM^SA/SA^* astrocytes (Figure 3E).

### 3.4. Mitomycin C Treatment or Serum Starvation Reduces the Fraction of Immature VIM^SA/SA^ Astrocytes with Vimentin Accumulations

To test whether the formation of vimentin accumulations is affected by cell proliferation, we added mitomycin C to the culture media or subjected the astrocytes to serum starvation—two different culture conditions that inhibit cell division. Cell proliferation as assessed by the fraction of cells positive for Ki67 was reduced by 84% and 74%, respectively (*p* < 0.00000001 for both). We observed that both mitomycin C and serum starvation led to a reduction in the fraction of immature *VIM^SA/SA^* astrocytes with vimentin accumulations (*p* = 0.000011 and 0.000122, respectively, Figure 4), which indicates that the presence of vimentin accumulations depends on cell proliferation.

### 3.5. The Fraction of Immature VIM^SA/SA^ Astrocytes with Abundant Vimentin Accumulations Is Reduced in the Absence of GFAP

Mature *VIM^SA/SA^* astrocytes contained lower levels of vimentin and higher levels of GFAP compared to mature *VIM^WT/WT^* astrocytes [47]. In cultures of immature *VIM^SA/SA^* and *VIM^WT/WT^* astrocytes, western blot analysis showed low but comparable protein levels of GFAP (*p* = 0.328) and comparable levels of nestin (*p* = 0.889, Figure 5A), while the levels of vimentin were lower in immature *VIM^SA/SA^* vs. *VIM^WT/WT^* astrocytes (*p* = 0.00002, Figure 5A). To test the hypothesis that GFAP compensates for the effect of *VIM^SA/SA^*, we crossed the *VIM^SA/SA^* mice with mice carrying a null mutation in the GFAP gene (*GFAP^−/−^*, [48]). *VIM^SA/SA^GFAP^−/−^* mice were viable, reproduced normally, and did not show any obvious phenotype. Compared to *VIM^SA/SA^* astrocytes, immature *VIM^SA/SA^GFAP^−/−^* astrocytes showed a reduction in the cell fraction with abundant vimentin accumulations (> 15 accumulations per cell, *p* = 0.016, Figure 5C). The fraction of bi-nucleated cells was not altered in *VIM^SA/SA^GFAP^−/−^* compared to *VIM^SA/SA^* immature astrocytes (*p* = 0.125, Figure 5D). We observed no difference in the fraction of bi-nucleated cells in mature *VIM^SA/SA^* and *VIM^SA/SA^GFAP^−/−^* astrocyte cultures (data not shown).

### 3.6. Immature VIM^SA/SA^ and VIM^SA/SA^GFAP^−/−^ Astrocytes Have Normal Mitochondrial Membrane Potential

**Figure 6:**
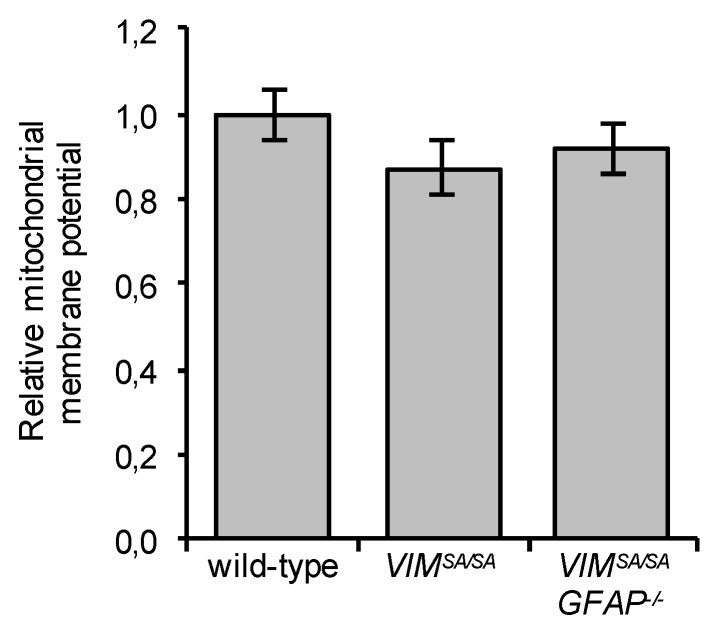
Immature *VIM^SA/SA^* and *VIM^SA/SA^GFAP^−/−^* astrocytes have normal mitochondrial membrane potential. The mitochondrial membrane potential was measured by using the TMRE probe. Immature wild-type, *VIM^SA/SA^*, and *VIM^SA/SA^GFAP^−/−^* astrocytes were incubated for 2 h in PBS and 30 min with TMRE. N = 7, 7, and 6, respectively.

Given the striking co-localization of mutant vimentin accumulations with mitochondria, we compared the mitochondrial membrane potential of wild-type, *VIM^SA/SA^,* and *VIM^SA/SA^GFAP^−/−^* astrocytes. We did not find any difference between these cells in the mitochondrial membrane potential as assessed by using the mitochondrial probe TMRE (*p* = 0.16 for *VIM^SA/SA^* and *p* = 0.34 for *VIM^SA/SA^GFAP^−/−^*, Figure 6).

### 3.7. Immature VIM^SA/SA^ and VIM^SA/SA^GFAP^−/−^ Astrocytes Show Normal Vulnerability to H_2_O_2_, Oxygen/Glucose Deprivation, and Chemical Ischemia

**Figure 7:**
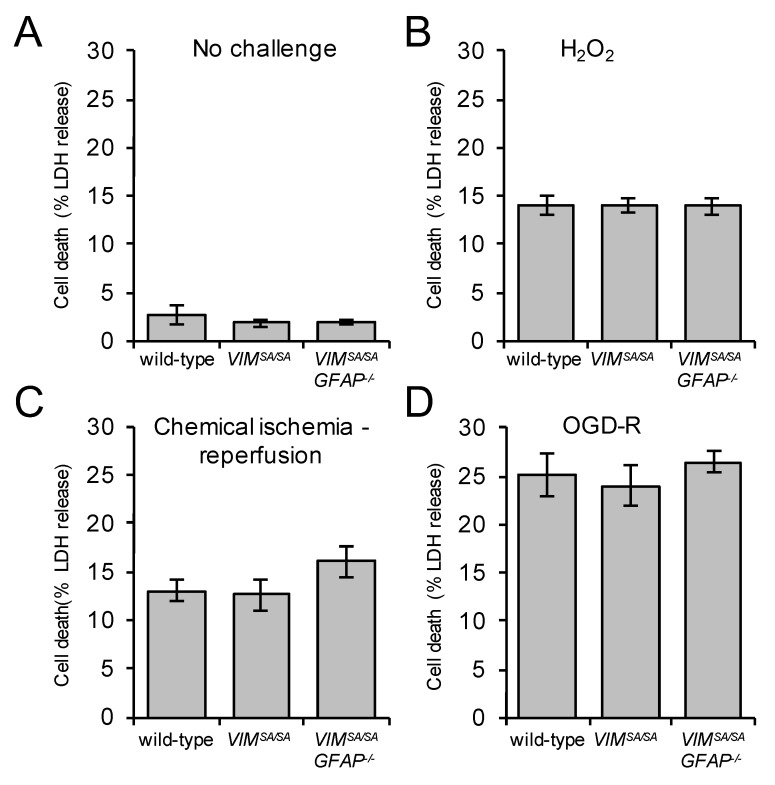
*VIM^SA/SA^* and *VIM^SA/SA^GFAP^−/−^* immature astrocytes show normal vulnerability to H_2_O_2_, chemical ischemia, and oxygen/glucose deprivation. Immature wild-type*, VIM^SA/SA^,* and *VIM^SA/SA^GFAP^−/−^* astrocyte cultures were incubated in (**A**) normal media with the B27 supplement to replace serum (no challenge), (**B**) exposed to H_2_O_2_ (500 µM) in HBSS buffer for 2 h, (**C**) exposed to chemical ischemia for 2 h followed by 2 h of reperfusion, or (**D**) exposed to oxygen/glucose deprivation for 18 h followed by 2 h of reperfusion (OGD-R). Cell death was measured as percentage of LDH release. N = 9, 5, and 10, respectively.

Astrocytes deficient in cytoplasmic intermediate filaments are more susceptible to cell death induced by oxygen and glucose deprivation followed by reperfusion (OGD-R), and chemical ischemia followed by reperfusion [7,57]. To assess the effect of *VIM^SA/SA^* on astrocyte survival after stress, wild-type, *VIM^SA/SA^,* and *VIM^SA/SA^GFAP^−/−^* astrocytes were exposed to H_2_O_2_, chemical ischemia followed by reperfusion or OGD-R. We did not find any difference in the extent of cell death assessed as the LDH release ((Figure 7A) *p* = 0.510 and 0.369, (Figure 7B) *p* = 0.988 and 0.923, (Figure 7C) *p* = 0.834 and 0.135, and (Figure 7D) *p* = 0.735 and 0.599), indicating that *VIM^SA/SA^* does not affect the resilience of astrocytes.

## 4. Discussion

Mutation of the vimentin serine residues phosphorylated during mitosis was previously shown to lead to bi-nucleation, cytokinetic failure, aneuploidy, and induction of senescence-related genes in lens epithelial cells [45]. In standard mature *VIM^SA/SA^* astrocyte cultures (14 days in vitro), the intermediate filament network is well developed and the astrocytes show normal cell proliferation [47]. Vimentin protein levels are decreased and GFAP protein levels are increased in mature *VIM^SA/SA^* astrocytes [47], pointing to a possible compensatory effect of GFAP expression. Here we addressed this putative compensation by GFAP using two different strategies.

First, we studied immature astrocytes, which express relatively low levels of GFAP. While the mutant vimentin protein levels were reduced in immature astrocytes, similar to mature astrocytes [47], the protein levels of GFAP in immature *VIM^SA/SA^* astrocytes were not altered. Interestingly, immature *VIM^SA/SA^* astrocyte cultures showed increased numbers of bi-nucleated cells, a finding suggestive of a cytokinetic failure. In more than 50% of immature *VIM^SA/SA^* astrocytes, we detected vimentin accumulations. These accumulations co-localized with mitochondria, were most prominent in *VIM^SA/SA^* astrocytes with low or no GFAP expression, but absent in *VIM^WT/WT^* astrocytes. The findings that 75% of bi-nucleated cells in the cultures of immature *VIM^SA/SA^* astrocytes contained vimentin accumulations and that the fraction of immature *VIM^SA/SA^* astrocytes with vimentin accumulations was reduced when cell proliferation was inhibited by serum starvation or mitomycin C treatment, further support the contention of a possible causal link between the *VIM^SA/SA^* mutation and the cytokinetic failure.

To further test the hypothesis that GFAP expression compensates for *VIM^SA/SA^*, we generated *VIM^SA/SA^GFAP^−/−^* mice. Cultures of immature *VIM^SA/SA^GFAP^−/−^* and *VIM^SA/SA^* astrocytes showed similarly increased numbers of bi-nucleated cells and both cultures contained astrocytes with vimentin accumulations. Surprisingly, immature *VIM^SA/SA^GFAP^−/−^* astrocytes showed 2.4 times lower number of cells with abundant vimentin accumulations (> 15 vimentin accumulations/cell). Thus, the complete removal of GFAP in immature *VIM^SA/SA^* astrocytes did not aggravate the phenotypic alterations of these cells, indicating that GFAP does not compensate for the effects of mutant vimentin.

Vimentin aggregates are found in pathological situations such as cataract induced by mutant vimentin [58], giant axonal neuropathy [59], or glyoxal-treated skin fibroblasts [60]. These aggregates are often found in a juxtanuclear or perinuclear position. In contrast, the vimentin accumulations observed in immature *VIM^SA/SA^* astrocytes were smaller and evenly dispersed. Moreover, these accumulations were not visible in mature astrocytes, indicating that with time in culture they disassemble, are degraded, or integrate into the intermediate filament network. So rather than representing a pathological aggregate accumulation, they might represent a transient stage in the dynamics of vimentin intermediate filaments that becomes accentuated or prolonged in immature *VIM^SA/SA^* astrocytes, but disappears with time.

Astrocyte intermediate filaments sense oxidative stress [61,62] and confer resilience to oxygen/glucose deprivation [7]. The extent of cell death induced by H_2_O_2_, oxygen/glucose deprivation, and chemical ischemia was comparable in *VIM^WT/WT^*, *VIM^SA/SA^,* and *VIM^SA/SA^GFAP^−/−^* astrocytes, indicating that the serine residues of vimentin phosphorylated during mitosis do not play a role in astrocyte resilience to oxidative stress.

The physical connection between mitochondria and vimentin intermediate filaments in mesenchymal cells was reported to increase mitochondrial membrane potential [63]. While co-localization of punctate vimentin accumulations with mitochondria in *VIM^SA/SA^* astrocytes was remarkably high, the co-localization with well-developed bundles of intermediate filaments was relatively low both in *VIM^SA/SA^* and *VIM^WT/WT^* immature astrocytes. We did not observe a difference in mitochondrial membrane potential between *VIM^WT/WT^*, *VIM^SA/SA^,* and *VIM^SA/SA^GFAP^−/−^* astrocytes. This might indicate that vimentin accumulations in *VIM^SA/SA^* astrocytes do not affect mitochondrial energetics or that the ceiling for vimentin-dependent activation of mitochondria was reached. We cannot rule out the possibility that mutant vimentin in the form of accumulations altered aspects of mitochondrial function other than mitochondrial potential.

In summary, we show that immature *VIM^SA/SA^* astrocytes contain vimentin accumulations that disappear with time in culture, are enriched in the fraction of bi-nucleated cells, and depend on cell proliferation. These vimentin accumulations co-localize with mitochondria, but do not seem to affect mitochondrial membrane potential. Immature *VIM^SA/SA^* astrocytes show normal resilience to oxidative stress. The combination of the *VIM^SA/SA^* and *GFAP^−/−^* mutations indicates that GFAP does not compensate for the effects of the *VIM^SA/SA^* mutation.
